# Gas Sorption Measurements in MOFs—A Tutorial for Beginners

**DOI:** 10.1021/acsmaterialsau.6c00063

**Published:** 2026-05-16

**Authors:** Marzena Pander, Chiranjib Gogoi, Wojciech Bury

**Affiliations:** † Faculty of Chemistry, 201871 Jagiellonian University, Gronostajowa 2, 30-387 Kraków, Poland; ‡ Faculty of Chemistry, 49572 University of Wrocław, F. Joliot-Curie 14, 50-383 Wrocław, Poland

**Keywords:** adsorption, porosity, isotherm, pore volume, BET analysis, metal−organic frameworks, MOF, sample activation, tutorial, data reporting

## Abstract

Adsorption measurements belong to the primary characterization tools used to determine the textural properties of porous solids such as Brunauer–Emmett–Teller (BET) area, pore volume, and pore size distribution as well as to estimate the energy of host–guest interactions. Accurate description of gas sorption in metal–organic frameworks (MOFs) is crucial for evaluating their performance and provides key insights into textural properties such as adsorption capacity, pore volume, and pore size distribution. This tutorial paper aspires to provide a practical guide for graduate and undergraduate students, as well as researchers who are new to the field, on low-pressure gas adsorption measurements (up to atmospheric pressure). Key aspects such as sample preparation, activation strategies, adsorption measurement techniques, data analysis, and reporting are discussed in detail. We believe that this Tutorial could serve as an entry-level teaching resource for gas sorption measurements in MOFs.

## Graphical abstract

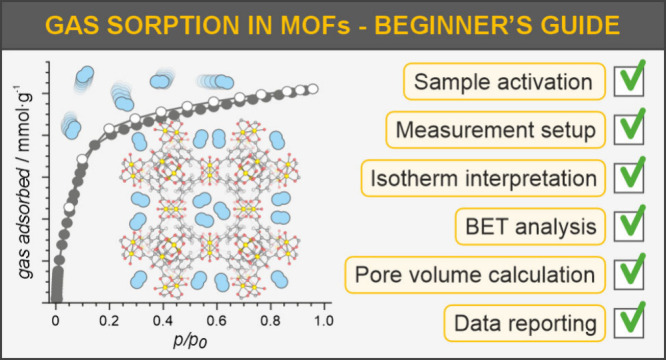

## 1 Introduction

Adsorption is defined as a surface phenomenon in which molecules of a substance present in the mobile phase (adsorbate) accumulate on the surface of a solid material, known as the stationary phase (adsorbent).^1,2^ The adsorption process is governed by intermolecular interactions between the adsorbate molecules and the surfaces of the adsorbent. These intermolecular interactions are similar to those responsible for the nonideal behavior of real gas and vapor condensation. Along with attractive dispersion forces and short-range repulsive interactions, specific molecular interactions such as polarization, field-dipole, and field gradient-quadrupole interaction may occur because of geometric and electronic characteristics of both the adsorbent and adsorbate.^3^ The reverse process to adsorption is desorption, in which the adsorbed molecules are released from the surface of an adsorbent back into the gas or liquid phase. From the thermodynamic point of view, adsorption processes are influenced by changes in pressure, temperature, and intermolecular interactions. According to the Le Chatelier principle, the direction of equilibrium will shift in that direction where stress can be relieved. In the case of pressure changes, increasing the pressure shifts the equilibrium toward the adsorbed phase, as this reduces the number of molecules in the gas phase. Consequently, a higher pressure generally favors adsorption. Temperature is another factor that affects the adsorption process, as adsorption is typically exothermic in nature and is therefore favored at lower temperatures.

Physisorption and chemisorption are two types of similar phenomena, with physisorption being a physical process driven by van der Waals forces and chemisorption involving the formation of chemical bonds (Figure 1).^4^ Both processes are exothermic in nature, increase with the expansion of the surface area, and both are associated with a decrease in entropy. The principal difference between the two processes is that chemisorption can only occur in a monolayer on a surface, which is directional, irreversible, and strong; while physisorption usually has multilayer adsorption, it is weak, nondirectional, reversible, and nonspecific, depending upon the temperature. The International Union of Pure and Applied Chemistry (IUPAC) classified pores in materials as follows: pores with widths below 2 nm are defined as micropores, those in the range of 2–50 nm as mesopores, and those greater than 50 nm are referred to as macropores.^5,6^ This classification is particularly useful when interpreting adsorption isotherms and evaluating the textural properties of porous materials.

**Figure 1:**
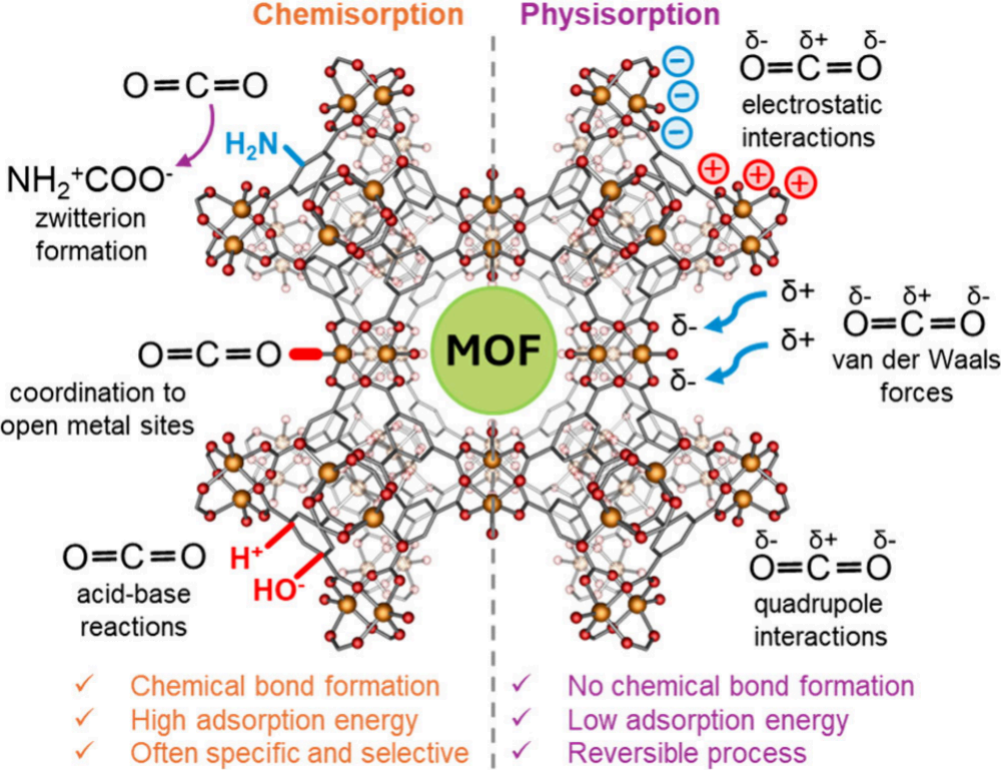
Adsorption mechanisms of CO_2_ in MOFs involving physi- and chemisorption.

Gas adsorption measurements play an important role in evaluating the performance of porous materials in applications such as gas storage, separation, catalysis and pharmaceuticals. A number of key parameters, such as adsorption capacity, pore volume, specific surface area and heat of adsorption are commonly used to characterize different solid materials, such as industrial adsorbents, catalysts, ceramics and porous coordination polymers.^7^ However, significant inconsistencies have been observed in how adsorption experiments are conducted, analyzed, and reported. Variations in sample preparation, “activation” procedures, equilibration criteria, and data fitting methods can lead to discrepancies in reported adsorption capacities even for identical material.

In this Tutorial, we discuss best practices and techniques covering sample preparation, activation procedures, adsorption measurements, and data analysis with a view of providing practical guidance for obtaining accurate and reproducible gas sorption data with special emphasis on metal–organic frameworks (MOFs).

The adsorption process is best understood through graphical representations called adsorption isotherms,^2^ which describe the relationship between the amount of adsorbate (*q*) taken up by a particular mass of adsorbent (*q*/*m*) at the equilibrium pressure (for gases) and at constant temperature. In order to describe this behavior, a number of theoretical adsorption models have been developed, including Henry,^8^ Freundlich,^9^ Langmuir,^10^ and the Brunauer–Emmett–Teller (BET) isotherm.^11^ On the basis of the shapes of the isotherms, IUPAC classified adsorption isotherms into six types (I–VI, Figure 2), each reflecting specific textural and surface characteristics.^12^

**Figure 2:**
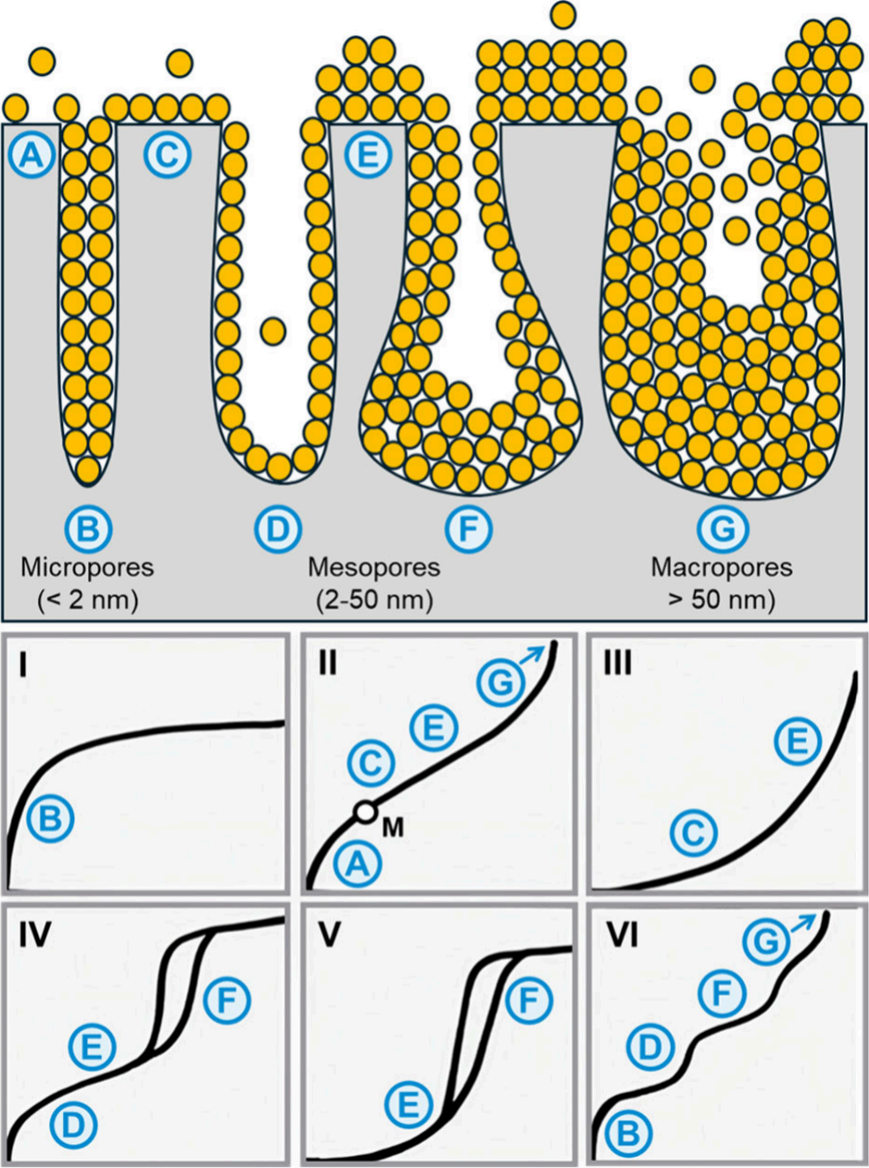
IUPAC classification of adsorption isotherms (bottom) and their relationship with the surface features (top). The schematic illustrates gas adsorption scenarios in microporous, mesoporous and macroporous materials, including micropore filling, monolayer-multilayer adsorption and capillary condensation within the different pore sizes.

The reversible type I isotherm (commonly called the Langmuir isotherm) is typically a result of monolayer adsorption. In case of physisorption, it occurs in microporous solids, where adsorption is dominated by micropore filling at low relative pressures (A–C, Figure 2). Once the micropores are filled, adsorption reaches saturation and further pressure increase causes only a small additional uptake. Type I is further divided into two subclasses, Type I­(a) and Type I­(b). Type I­(a) isotherms are given by materials with mostly narrow micropores (pore width ≲1 nm), while Type I­(b) isotherms are given by materials with a broader pore size distribution including wide micropores and potentially narrow mesopores (pore width up to ≲2.5 nm).^3^ A Type II isotherm typically occurs in case of nonporous or macroporous adsorbents, where unrestricted multilayer formation can occur. The M point shown in isotherm II represents the completion of the monolayer before the adsorption in the second and subsequent layers begins (A, C and E, Figure 2). Type III isotherm is convex to the relative pressure axis over its entire range and subsequent layer formation begins before completion of the first layer (E, Figure 2). In this case, the attractive interactions of adsorbate–adsorbent are relatively weak in comparison to interactions between adsorbate–adsorbate. A Type IV isotherm is characteristic of mesoporous solids and exhibits features related to capillary condensation within the mesopores. This isotherm is an extension of a Type II isotherm, where in this case adsorption reaches saturation point in a subsequent layer (D–F, Figure 2). The most characteristic feature of a Type IV isotherm is the hysteresis loop, which is caused by the filling of mesopores. Type IV isotherms are subdivided into Type IV­(a) and Type­(b). Type IV­(a) exhibits a well-defined hysteresis loop due to capillary condensation within mesopores (2–50 nm), while Type IV­(b) shows little or no hysteresis loop, because the radius of the mesopores is smaller than the critical radius for the occurrence of hysteresis.^13^ A Type V isotherm is distinguished by its characteristic S-shape, and it also shows pore condensation and the hysteresis loop. The initial part of the isotherm is related to Type III, where attractive interactions between adsorbent–adsorbate are relatively weak (E, Figure 2). Type VI describes a more complex scenario where all different types of surface features can be present in the sample (A–G, Figure 2). Such behavior is less common in practical situations. All the isotherms have inflection points which represent the change of the gas–solid interaction force or gas–gas interaction force that occurred between different adsorption processes.^14^

The experimental methods to determine gas adsorption isotherms are mainly categorized as *volumetric* (manometric) and *gravimetric* methods.^15^ In volumetric experiments, the adsorption uptake is determined by measuring the pressure change of a known quantity of gas in a calibrated volume after it has been exposed to the adsorbent. In contrast, gravimetric techniques directly measure the change in the sample mass resulting from adsorption of guest molecules.

In most adsorption experiments, the measured quantity is the surface excess amount, which describes how many molecules were adsorbed at the surface in excess of what had been available from the bulk gas phase. In both the volumetric and gravimetric methods, we can obtain only the excess gas adsorption amount (Gibbs excess) rather than the absolute (true) adsorption amount.^16^ The surface excess gas molecules are those molecules that are directly adsorbed onto the surface, whereas absolute gas molecules include the gas molecules adsorbed on the pore surface and those occupying the pore volume as compressed gas. The experimentally measured excess adsorption is converted to the total adsorption amount by using Gibbs definition: *N*_ex_ = *N*_tot_ – *d*_gas_ *V*_pore_, where, *N*_ex_ and *N*_tot_ are the excess and total uptake amounts respectively, mmol g^–1^; *d*_gas_ is the density of the gas at a given pressure in g cm^–3^, and *V*_pore_ is the pore volume of the adsorbent in cm^3^ g^–1^.^17^ In low-pressure measurements (up to atmospheric pressure), the reported adsorption is typically expressed as the *excess adsorption*, which differs negligibly from the *absolute adsorption*. At higher pressures, however, this distinction becomes significant because of high density of the gas at pressure above 1 atm, and the adsorption basis should always be clearly specified. For measurements conducted under conditions where the gas phase deviates significantly from ideal behavior, *fugacity* rather than pressure should be used; this consideration is generally not critical for the low-pressure measurements discussed in this tutorial.

Over the past few years, MOFs have emerged as one of the most widely studied classes of porous materials due to their exceptionally high surface areas, structural control over the framework, and customizable framework functionalities.^18^ Since the pioneering early contributions to the development of MOFs, the field has expanded rapidly, and they have been extensively studied for applications in gas storage, carbon capture, separations, catalysis, sensing, etc.^19,20^ Among these applications of MOFs, they are particularly important for their ability to selectively adsorb a wide range of gases or vapors. The modular design of MOFs allows a rational control over pore size, topology, and chemical functionality—while functionalized linkers and open metal sites, along the pore surfaces, provide selective recognition sites for gas molecules (Figure 1).^21–23^

## 2 Before the Measurement: Sample Preparation and Activation

Accurate gas adsorption measurements begin not with the instrument, but with proper sample preparation. One of the most key challenge lies in their “activation” after synthesis.^24^ The activation of a MOF sample is defined as a process of removal of guest molecules from the MOF material without compromising its structural integrity and hence porosity.^25^ Incomplete activation is one of the most common sources of irreproducible adsorption data. Typically, it also leads to the pore structure becoming inaccessible and making the material unusable.

A recommended workflow of a sorption experiment for an MOF sample is illustrated in Chart 1, where the key steps, from the synthesis to data reporting, are shown. The process starts with the synthesis and isolation of the MOF sample, followed by the verification of its structural integrity (before and after solvent removal). Next, an appropriate activation strategy should be selected, and its completeness should be confirmed. Afterward, adsorption measurement can be performed under selected conditions. The acquired isotherm data can be subsequently analyzed, and the structural stability of the material should also be verified after the sorption experiments. Finally, the adsorption results are reported together with the relevant experimental details to ensure reproducibility and proper interpretation, as discussed in the following sections.

**Chart 1:**
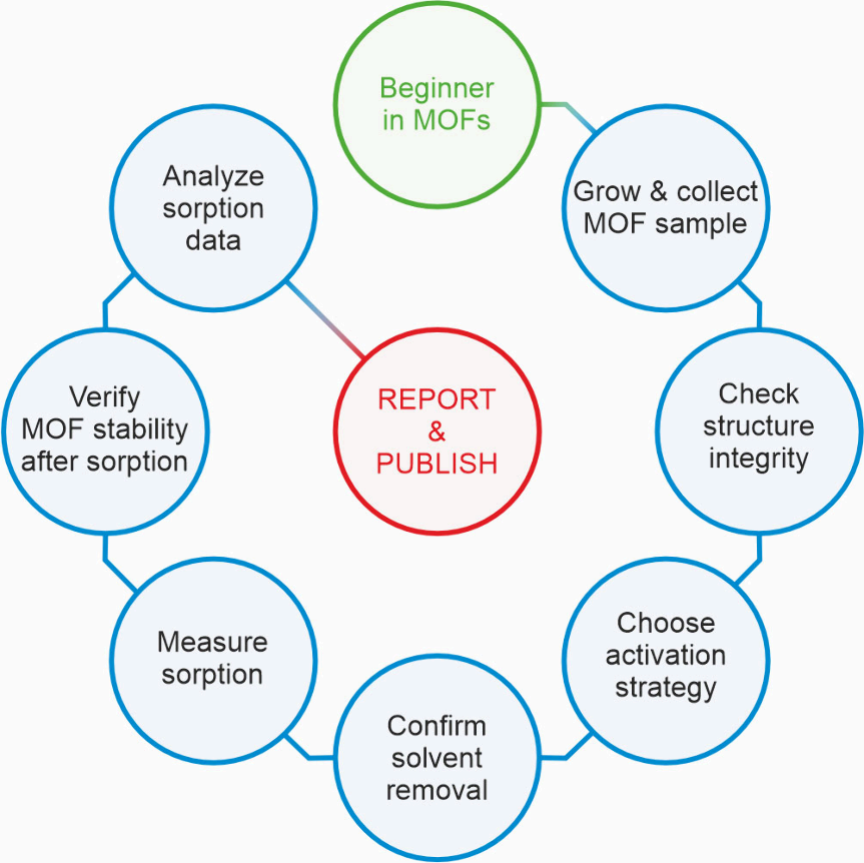
Workflow for a Sorption Experiment on a MOF Sample, Showing the Key Steps from Sample Preparation and Activation through Adsorption Measurement, Data Analysis, and Reporting

### 2.1 Activation Process of a MOF Sample

In general, MOFs are typically synthesized in polar, high-boiling solvents such as *N,N*-dimethylformamide (DMF), *N,N*-diethylformamide (DEF), *N,N*-dimethylacetamide (DMA), *N*-methyl-2-pyrrolidone (NMP), etc. The guest molecules include solvents or other compounds used or formed during the synthesis of a MOF. Some unbound ligands tend to bind to the metal nodes during synthesis, which obstruct the pores and render them inaccessible to gas molecules. Therefore, removing these extra guest molecules is a crucial procedure that helps to not only make it porous but also generate open metal sites and enable gas molecules to bind effectively. Typical activation methods are discussed briefly in the following paragraphs.^26^

#### 2.1.1 Conventional Heating and Vacuum

This is the simplest and most used method applied for removal of guest molecules from the pores of the MOF. In this approach, MOF material is heated in the temperature range 80–300 °C for several hours under dynamic vacuum to evaporate guest molecules. Some examples of MOFs which use this method for activation are Cr-MIL-101, UiO-66, etc.^27,28^ However, many other MOFs cannot tolerate this approach, often experiencing a complete loss of crystallinity after activation. This structural collapse may be attributed to the fact that the solvent within the pore undergoes liquid to gas phase transition, which generates significant surface tension and capillary stress that exceeds the relatively moderate metal–ligand coordination bond strength in less stable MOFs.

#### 2.1.2 Solvent-Exchange

This activation method involves replacing a high boiling solvent such as DMF, DEF, or DMA with a more volatile solvent like methanol, acetone, chloroform (CHCl_3_), tetrahydrofuran (THF), dichloromethane (DCM), or dimethyl ether, followed by gentle heating under vacuum. Although low-boiling solvents are used for solvent exchange, the effectiveness of the solvent exchange process is also related to the surface tension of the solvent. Solvents with lower surface tension generate smaller capillary stress during desolvation, thereby minimizing possible structural changes or framework collapse. A solvent like perfluorohexane, despite its relatively high boiling point, is known to provide gentle activation compared to DCM due to its exceptional ultralow surface tension.^29^ After obtaining the synthesized material, MOF should be initially washed thoroughly with the original synthesis solvent to remove any uncoordinated linkers and residual impurities. For solvent exchange, MOF should be left to soak in the low or ultralow surface tension solvents. It often requires extended soaking times ranging from hours to several days to ensure that the high-boiling solvent is fully replaced. Sometimes, solvent exchange can also be performed by a Soxhlet extraction where a low-boiling point solvent is used as an extractant, for example in the case with UiO­(bpdc) (bpdc = 2,2-bipyridine-5,5′-dicarboxylate).^30^

#### 2.1.3 Supercritical CO_2_ (scCO_2_) Processing

The use of scCO_2_ is another method of activation of MOFs. It is considered as a milder activation approach with solvent-exchange extension where scCO_2_ is used rather than solvents like CHCl_3_ or THF for DMF.^31^ scCO_2_ is a good option for activation because it is nontoxic in nature, inexpensive, and easy to scale up and also has low surface tension. In contrast to solvent-exchange and conventional thermal activation methods, which may induce partial or complete framework collapse, activation using supercritical CO_2_ is considered a gentler approach and less destructive technique. During scCO_2_ treatment, the solvent is removed under supercritical conditions, thereby avoiding the liquid–gas phase transition. Because this process eliminates surface tension and capillary stress, typically generated at the liquid–vapor interface, the structural integrity of the MOF is better preserved. BET surface area of IRMOF-3 was found to be 2850 m^2^ g^–1^ after being activated with scCO_2_, which represents a 1.6-fold increase over the solvent exchange (1800 m^2^ g^–1^).^32^ The BET surface area of IRMOF-16 was reported as 1910 m^2^ g^–1^, which represents a 4-fold increase over the same material when activated by solvent exchange.^33^

#### 2.1.4 Freeze-Drying

In this approach, as-synthesized MOF is first subjected to solvent exchange with a suitable low freezing-point solvent such as benzene and cyclohexane to replace high boiling solvents. The sample is then frozen and solvent is removed by sublimation under reduced pressure.^34^ Since solvent transitions directly occur from the solid to the vapor phase, the liquid–gas interface is avoided. This significantly reduces associated capillary forces within the pores and minimizes the risk of pore collapse or structural degradation. The BET surface area of FIR-3 MOF found to be only 24 m^2^ g^–1^ using a conventional solvent exchange method, but activation via cyclohexane-assisted drying produced a BET surface area of 497 m^2^ g^–1^.^35^

#### 2.1.5 Chemical Treatment

In certain cases, MOF activation requires chemical treatment, particularly when guest species are ionic or strongly coordinated high-boiling solvents. These molecules are difficult to remove by simple heating because of their low volatility or strong coordination to metal nodes. In such cases, acid treatment or coordination exchange processes are employed to displace bound modulators or solvent molecules from the framework. Zirconium-based MOFs are especially suitable for this approach, because of their high chemical stability. For example, PCN-222 (also known as MOF-545) shows increased pore volume after treatment with HCl followed by activation under vacuum and heat.^36^ Similarly, in NU-1000, acid treatment removes coordinated benzoates from the Zr_6_ nodes, generating mesoporosity and exposing additional active sites.^37^

### 2.2 Verification of Complete Activation of the Sample

As noted above, complete activation of the synthesized MOFs is crucial to accessing the permanent porosity and high surface area. There are several techniques to verify the completeness of the activation process. Among them, mass monitoring, powder X-ray diffraction (PXRD), and thermogravimetric analysis (TGA) are the most important.

#### 2.2.1 Mass Monitoring

Measurement of the sample mass before and after activation provides an initial indication of solvent removal. The observed mass loss can be compared to the theoretical solvent content derived from crystallographic data or thermogravimetric analysis. However, some important limitations should be considered when interpreting mass loss data after activation. The decrease in the mass of the sample after activation does not distinguish between pore-filling solvent and partial decomposition of the framework. Strongly bound or coordinated solvent molecules may remain undetected if present in small quantities. For hygroscopic MOFs or those containing open metal sites, rapid transfer is recommended to minimize readsorption of water prior to the measurement.

#### 2.2.2 Powder X-ray Diffraction

PXRD remains one of the most reliable tools for verifying structural integrity after the activation process. Comparison of diffraction patterns before and after activation allows assessment of crystallinity and phase purity as well as possible structural transformations.^38^ Significant diffraction peak broadening may suggest partial amorphization; however, it should be noted that a quantitative assessment of amorphization is not straightforward without the use of a reference compound. The disappearance of the strongest reflections may be the result of the framework collapse, while shifts in the peak position can indicate the structural contraction or phase transitions after activation. In the case of flexible or breathing MOFs, change in the diffraction peak positions or disappearing or appearing of new peaks should be carefully analyzed.^39^ In such cases, comparison with simulated patterns of known phases is recommended before deciding whether observed changes arise from structural degradation or a reversible phase transformation.

#### 2.2.3 Thermogravimetric Analysis

TGA is another complementary analytical technique that can provide useful information on the presence of residual guest molecules in porous materials.^40^ When an activated sample is reheated in an inert atmosphere, one or more additional mass-loss steps may appear due to release of solvent molecules retained inside the pores after activation. However, sometimes TGA data can be difficult to interpret as both solvent desorption and framework decomposition may occur over similar temperature ranges. In some cases, strongly coordinated solvent molecules need much higher temperatures for removal than weakly physiosorbed guests located within the pores. Therefore, comparing the TGA profile of the activated material to that of the as-synthesized sample can be useful for detecting an incomplete activation.

Apart from these techniques, Fourier transform infrared (FT-IR) spectroscopy^38^ and nuclear magnetic resonance (NMR)^41^ also helps to identify the complete activation process of the sample. For example, in the case of FT-IR, characteristic peaks of residual solvent molecules or unreacted ligands should disappear from the activated sample. For instance, the stretching vibration of a carbonyl group from the DMF molecule is observed at around 1652 cm^–1^; this peak should be absent for the activated sample.^42^ Similarly, solution-state ^1^H NMR of digested samples after activation can also confirm the presence or absence of guest solvent molecules within the frameworks also in a quantitative manner, e.g. DMF, ethanol, acetone or others; however, these solvents should contain hydrogen atoms to be detected in an ^1^H NMR experiment.^43^

## 3 Running the Adsorption Measurement

In a **volumetric adsorption** measurement, a known amount of pure adsorptive gas is dosed into a calibrated volume that contains the activated sample while maintaining a constant temperature. As the adsorption proceeds, the pressure of the gas decreases until equilibrium between the adsorbed phase and the gas phase is reached. The quantity of gas adsorbed (*q*) is then calculated from the difference between the initial amount of gas introduced and the amount required to fill the free space of the measurement system. This unoccupied part of the system volume, commonly termed *free space* or *dead volume*, is determined independently to enable accurate determination of the adsorption uptake.

In a typical experiment, the adsorption isotherm is obtained by sequentially dosing small quantities of gas into the measurement manifold. After each dose, the system pressure is monitored until equilibrium is reached according to the predefined criteria. The equilibrium pressure and the calculated amount adsorbed define one point on the adsorption isotherm, and repeating this procedure over a range of pressures generates the complete adsorption isotherm. The accuracy and reliability of adsorption measurements depend on the proper selection of experimental parameters and measurement conditions, which are discussed in the following section.

### 3.1 Selection of Adsorbate and Temperature Control

In sorption studies of MOFs and related porous materials, several adsorbates are commonly used depending on the target properties of the material. The primary characterization of these materials is performed using nitrogen (at 77 K) or argon (at 87 K), with N_2_ adsorption being the most reported in the literature due to the easier accessibility of liquid nitrogen.^44^ Using the adsorptive close to its normal boiling point allows the relative pressure range to be accessed up to the saturation pressure, which is necessary to characterize the full pore-size range, from ultramicropores to large mesopores. Argon adsorption is often recommended instead of nitrogen adsorption because argon does not possess a quadrupole moment and therefore minimizes specific interactions with polar adsorbents.^45^ The characterization of the material can be further extended to functional studies with more specific gases such as carbon dioxide, hydrogen, methane, or vapors (e.g., water, alcohols, or alkanes).^46^ The sorption of CO_2_ is typically performed either at 195 K or at near-ambient temperatures (273–298 K) and is often used to probe the porosity of microporous or flexible materials that may not show significant uptake of N_2_ at 77 K.^47^ Carbon dioxide might be also a very useful adsorbate in this context, due to its smaller kinetic diameter (3.30 Å compared with 3.64 Å for N_2_), which allows access to narrower pore apertures that are less accessible to N_2_ at 77 K. The purity of adsorptive gas or vapor is an important factor, as even trace impurities may compete for adsorption sites or accumulate in the pores of the material. Hence, high-purity gases (≥99.999% or 5N) and properly degassed high-purity solvents should be employed.

The adsorption temperature is selected based on the physical properties of the adsorbate, and its accurate control is essential for reliable adsorption studies. For measurements with N_2_, the sample cell is typically immersed in a cryogenic bath containing liquid nitrogen (77 K). Argon adsorption at 87 K can also be performed by using liquid argon or cryostats, where the latter provides a more cost-effective solution. For other adsorbates, the temperature is controlled using thermostabilized liquid baths (e.g., dry ice/isopropanol bath at approximately 195 K for CO_2_ adsorption) or temperature-stabilized circulating baths filled with appropriate coolant.

### 3.2 Sample Loading and Pretreatment

#### 3.2.1 Choice of the Sample Mass

The recommended mass of the sample for adsorption measurement depends on the expected surface area of the material. The adsorption uptake should be large enough to produce measurable pressure changes in the volumetric system but not so large that excessive dosing steps are required. If the uptake is too small, the resulting pressure change may be insufficient for accurate measurement. Conversely, very large adsorption capacities may require many dosing steps, which significantly prolongs the experiment. In practice, an approximate estimation of the expected surface area can help to adjust the sample mass. For example, for highly porous MOFs (>1000 m^2^ g^–1^), approximately 30–50 mg of the material would be enough to measure a full isotherm. For medium surface area samples, 50–100 mg is typically adequate, while low-porosity samples may require larger amounts of 100–200 mg. Importantly, very low sample masses should be avoided, when possible, as they can introduce substantial uncertainty from weighing and sample handling, particularly for lightweight, static-prone MOF powders. For samples with unknown expected porosity or materials that may undergo structural transformation during adsorption (e.g., gate opening in flexible MOFs),^48^ a practical starting point would be approximately 100 mg of degassed adsorbent sample.

#### 3.2.2 Loading Sample into Measurement Cell

After the required sample mass is selected, the material is loaded into the glass sample cell used for the measurement. Note that the sample should be placed at the bottom of the tube so that it is fully immersed in the temperature-controlled bath during the measurement. If the sample is positioned too high in the tube, then only part of the material may be analyzed at the adsorption temperature, which may lead to the underestimation of the adsorption capacity. As many MOFs are often obtained in powder form, the use of small funnels can facilitate loading of the sample into the cell. Depending on the cell design and its diameter, an additional filler rod can be inserted above the sample to reduce the free volume in the cell. In some cases, sample cells are also equipped with appropriate frits, caps, or sealing stoppers that help to prevent the loss of fine particles during handling and evacuation.

#### 3.2.3 Weighting Sample Cell

In practice, at least three mass measurements are required: (1) the empty sample cell, (2) the cell containing the sample before degassing, and (3) the cell with the sample after degassing. These measurements should be performed using a well-calibrated balance with a precision of at least four decimal places. After degassing, the sample cell should be allowed to cool to room temperature before being weighed to avoid any errors. It is also good practice to repeat weightings and report the average value to improve the reliability of the measured masses.

#### 3.2.4 Sample Pretreatment–Degassing Procedure

Prior to the adsorption experiment, the sample must undergo an additional pretreatment step in which residual gases or solvent molecules are removed from the pores of the material. This degassing procedure is performed either directly in the adsorption instrument (if equipped with a degassing station) or in a separate pretreatment device. Typically, in this process, the cell containing sample is heated under vacuum while the desorbed guest molecules are continuously evacuated from the system. The selected temperature should be high enough to remove the adsorbed guests but remain below the decomposition temperature of the material. Correlating the degassing temperature with TGA results (see details in section 2.2) can help to determine appropriate conditions. For various MOFs, degassing temperatures in the range of 80–150 °C are commonly applied.

The required degassing time should be adjusted to the studied material, sample amount, and type of guest molecules to be removed. Complete degassing should be verified by suitable criteria, such as the outgassing pressure rate or final pressure in the sample. For high-resolution low-pressure measurements, direct *in situ* degassing on the instrument is often preferable because it minimizes sample exposure to air and allows evacuation under the conditions required for the selected measurement program. Many systems also allow the user to define heating ramps and pressure changes during the degassing procedure, which may help to avoid rapid desorption or powder elutriation. The leak-check and evacuation criteria should also be adapted to the measurement setup, especially for low-pressure and micropore analyses. Finally, recording the applied degassing temperature and time, heating rate, and pressure change (e.g., in the Supporting Information or AIF file) ensures reproducibility of the performed sorption studies.

### 3.3 Setting up the Adsorption Measurement

#### 3.3.1 Instrument Preparation

Before starting the adsorption experiment, the measurement system must be properly prepared and checked. Most modern volumetric adsorption instruments perform several automated procedures during system initialization, including, for example, leak testing and free volume measurement. A *leak test* is performed prior to the measurement to confirm the integrity of the vacuum system. The determination of the *dead volume* of the sample cell, which corresponds to the volume of gas not occupied by the adsorbent, is typically done automatically using helium as a nonadsorbing probe gas.^46^ However, for ultramicroporous materials and high-resolution low-pressure measurements, helium may partially adsorb or become trapped at cryogenic temperatures, which can introduce errors.^3^ In such cases, a longer outgassing time after He exposure, the use of calibrated sample cells, or dead volume determination after the sorption measurement should be considered. Similarly, parameters describing commonly used adsorbates, including nonideality correction factors and adsorbate properties used in uptake calculations, are typically implemented in the software and can be verified when setting up the measurement. When comparing these values, it should be noted that the adapted units for pressure, adsorption uptake, and adsorbate parameters may differ between instruments. These instrument-specific details are provided in the manuals of the respective devices.

#### 3.3.2 Pressure Range and Distribution of Measured Points

The pressure range used in adsorption measurements depends on both the adsorbate and the capabilities of the instrument. For subcritical adsorbates such as N_2_ or Ar, adsorption is typically reported as a function of relative pressure *p*/*p*_0_, where *p*_0_ corresponds to the saturation pressure of the adsorbate at the measurement temperature. In many instruments *p*_0_ is determined automatically during the experiment to account for small temperature variations and to avoid condensation when approaching saturation conditions. The minimum measurable pressure depends on the sensitivity and accuracy of the pressure transducers used in the instrument. Modern volumetric systems typically allow measurements down to relative pressures of approximately *p*/*p*_0_ ≈ 10^–6^–10^–7^ for N_2_ adsorption at 77 K, while the upper limit approaches atmospheric pressure (or the saturation pressure of the adsorbate).

Once the pressure range is defined, the number and distribution of measurement points must be selected. For standard nitrogen adsorption measurements at 77 K, typically 40–80 points are sufficient to obtain a reliable adsorption isotherm, although the optimal number depends on the expected porosity of the material. Denser sampling in the low-pressure region is particularly important for micropore analysis and for reliable determination of BET surface area. Additionally, measurement points may be distributed using logarithmic spacing at low pressures and more linear spacing at higher pressures. Some instruments also allow the registration of additional points when rapid changes in adsorption uptake occur, which can improve the resolution of steep adsorption steps in the isotherm (Figure 3a).

**Figure 3:**
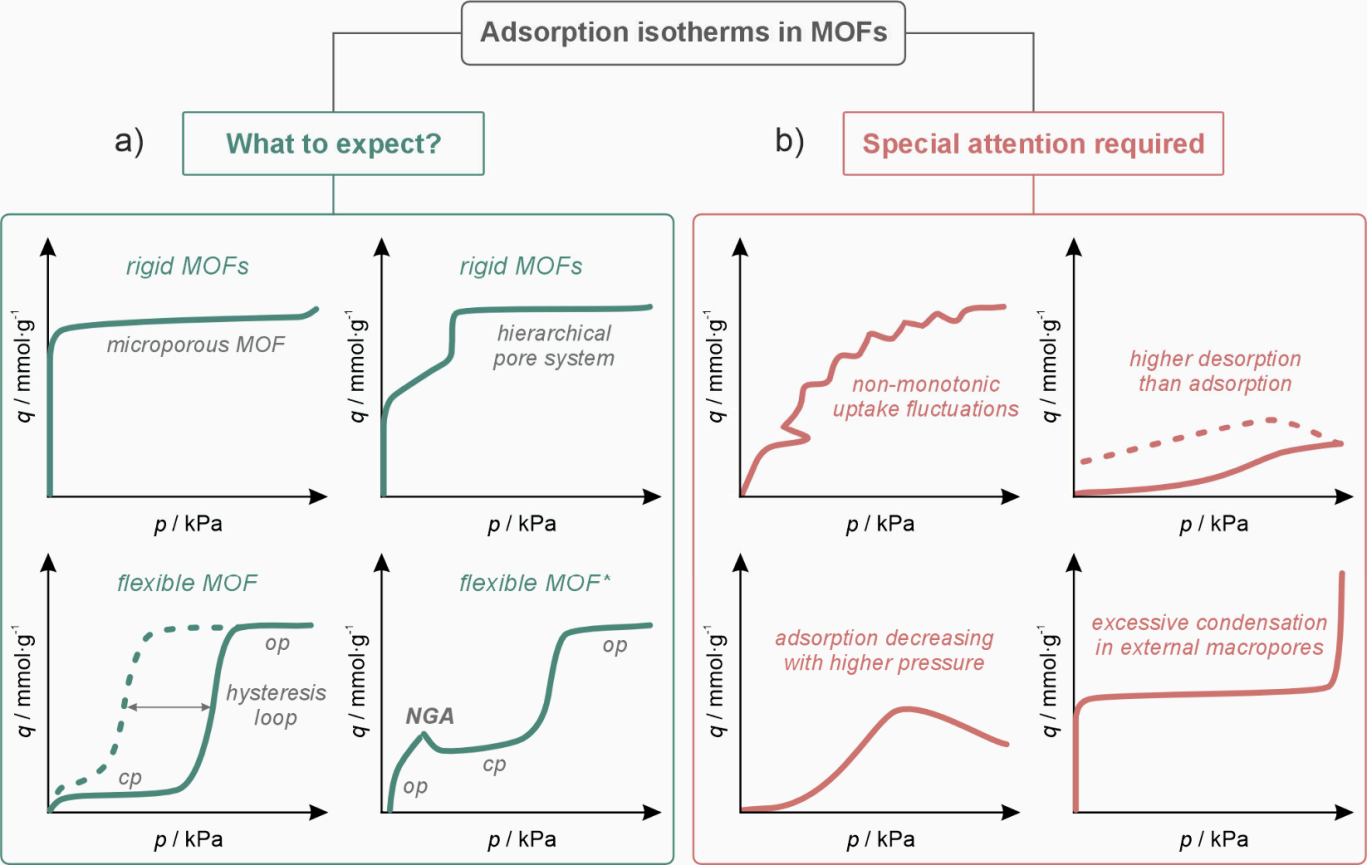
Schematic representations of adsorption isotherms: (a) typical adsorption behavior in MOFs, and (b) examples of isotherm shapes requiring special attention and verification of the measurement conditions. Solid lines represent adsorption branches and dotted lines represent desorption branches. For flexible MOFs, cp is close-pore phase, op is open-pore phase, and NGA is negative gas adsorption.

#### 3.3.3 Equilibration Criteria and Dosing Strategy

During an adsorption measurement, a gas is introduced into the sample cell in successive dosing steps until the targeted pressure points are reached. At each step, the system must reach an equilibrium before the adsorption uptake is recorded. Equilibration is typically determined using a pressure stabilization criterion where the change in pressure over a defined time interval must fall below a specified threshold. In practice, this criterion is usually combined with a minimum equilibration time to ensure that the system has a sufficient time to stabilize.

Most instruments allow the *equilibration criteria* to be adjusted depending on the pressure range. Manufacturers provide reference protocols for standard materials that can be used as a starting point for setting up measurements and further adjusted, if needed, for a particular material. Longer equilibration times are often required at very low pressures (e.g., below *p*/*p*_0_ ≈ 10^–4^), where adsorption proceeds slowly, while shorter equilibration times may be sufficient at higher pressures where adsorption uptake increases more rapidly. For materials exhibiting unusual adsorption mechanisms, such as flexible frameworks that undergo structural transitions during adsorption, the equilibration criteria may need to be adjusted to properly capture steep adsorption steps (Figure 3a).

In addition to equilibration criteria, the *dosing strategy* determines how gas is introduced and how measurement points are registered. Depending on the instrument software, parameters such as the pressure tolerance, dose size, or allowable pressure deviation can be defined. Although the terminology may vary between instruments, these parameters generally control the accuracy of the targeted pressure points and the rate of the data collection. If the tolerance is set too large, then the measurement may skip important pressure regions, while overly strict criteria may significantly prolong the experiment. Some instruments also allow for the registration of additional adsorption points when large changes in adsorption uptake are detected between successive measurements. This feature can improve the resolution of steep adsorption steps but may also increase the total measurement time.

Additionally, it is often advisable to perform a *preliminary test measurement* when studying a sample with unknown porosity. A short test isotherm using fewer points and shorter equilibration times can provide an initial estimate of adsorption uptake and help optimize the required sample mass, selection of number and distribution of pressure points, and equilibration criteria before performing the full measurement. The total duration of an adsorption experiment is therefore determined primarily by the number of measurement points and the equilibration criteria applied. For standard nitrogen adsorption measurements at 77 K, the total experiment time typically ranges from 24 to 72 h.

### 3.4 Adsorption Studies in MOFs and Common Experimental Pitfalls

Adsorption isotherms measured for MOFs can exhibit a wide variety of shapes that reflect their diverse pore structures and adsorption mechanisms. Representative schematic examples of adsorption isotherms commonly observed in MOFs are presented in Figure 3a. For MOFs that maintain a rigid structure after removal of guest molecules during the activation process, the observed adsorption behavior is primarily determined by the pore architectures of the material. In general, microporous MOFs exhibit a Type I adsorption isotherm (Figure 2), observed, for example, in the UiO-6x (x = 6,7,8) series.^28^ At the same time, mesoporous MOFs containing hierarchical pore structure are characterized by a Type IV adsorption isotherm, where a second steep uptake at higher relative pressure occurs. Examples of such behavior have been reported for Zr-based mesoporous MOFs such as NU-1000 ^37^ or PCN-222.^36^

For flexible MOFs that undergo stimuli-induced structural transformations, the adsorption mechanism is governed by the dynamic response of the framework to the applied stimuli, e.g. introduction or removal of guest molecules.^48^ In these systems, adsorption isotherms often exhibit distinct regions corresponding to different structural states of the material. For example, the closed-pore (*cp*) phase displays low adsorption uptake, while a structural transition occurring above a critical pressure leads to the formation of an open-pore (*op*) phase with significantly higher adsorption capacity (Figure 3a). During desorption, the reverse transition (*op* → *cp*) can occur at different pressures, resulting in a more pronounced hysteresis loop. These pressure-induced phase transitions can enhance the working capacity of the material for gas storage applications,^49^ or can contribute to selective adsorption behavior for specific adsorbates.^50^ A more unusual adsorption mechanism has been observed in certain flexible MOFs, such as DUT-49, which exhibits a negative gas adsorption (NGA) phenomenon.^51^ In this case, a pressure induced structural contraction of the framework results in the spontaneous release of previously adsorbed molecules despite an increase in external pressure.^52^

In contrast to the adsorption behavior described above, researchers new to adsorption measurements may encounter several practical problems when measuring adsorption in MOFs. Selected examples of such cases are schematically presented in Figure 3b. Careful examination of the isotherm shape can often provide useful diagnostic information regarding possible experimental issues. For example, when the monotonic increase of the adsorption branch is not maintained and the measured points show significant fluctuations, this might indicate that unstable experimental conditions. Possible causes include an insufficient equilibration time selected, uncertainties in dead volume determination, temperature fluctuations in the cooling bath, or pressure instability due to leaks in the system. Particularly, in cryogenic temperature measurement, attention should be paid to maintaining the bath level that ensures full immersion of the studied sample throughout the total time of the running experiment.

Another commonly encountered artifact is the appearance of a desorption branch above the adsorption branch or an apparent decrease in the adsorption uptake with increasing pressure (Figure 3b). Such behavior is thermodynamically inconsistent and should prompt verification of the measurement setup. This may occur when the sample has very low porosity or when an insufficient amount of material is used for the measurement because small adsorption uptakes are more strongly affected by uncertainties in dead volume determination. Similar artifacts may also result from incomplete equilibration or from the use of inappropriate nonideality correction factors. Broad hysteresis with a desorption branch showing a positive slope may indicate slow adsorption kinetics or diffusion limitations. Therefore, for materials with narrow pore apertures, increasing the equilibration time may not fully resolve the issue. In such cases, an alternative probe molecule with a smaller kinetic diameter, such as CO_2_ measured at 195 K, may be more appropriate than N_2_ measured at 77 K. Finally, an excessive uptake observed close to saturation pressure (*p*/*p*_0_ > 0.98) is represented by a sharp increase on the isotherm (Figure 3b). This effect can result from gas condensation in interparticle macropores or external void spaces in the system, which does not reflect the intrinsic porosity of the material and should be interpreted with caution.

## 4 After the Measurement: Data Reporting and Analysis

### 4.1 How to Present Adsorption Isotherm?

The results of sorption analysis are typically presented as an adsorption isotherm, i.e., the amount of adsorbed species (*q*) plotted as a function of pressure (*p*) at constant temperature (Figure 4). The visualization of an isotherm is not merely a matter of aesthetics, but the appropriate choice of axes, units, and overall graphical representation can directly affect clarity and reproducibility of the analyzed data. The recommendations for physisorption of gases (and the evaluation of surface area and pore size distribution) are provided in an IUPAC technical report from 2015.^3^

**Figure 4:**
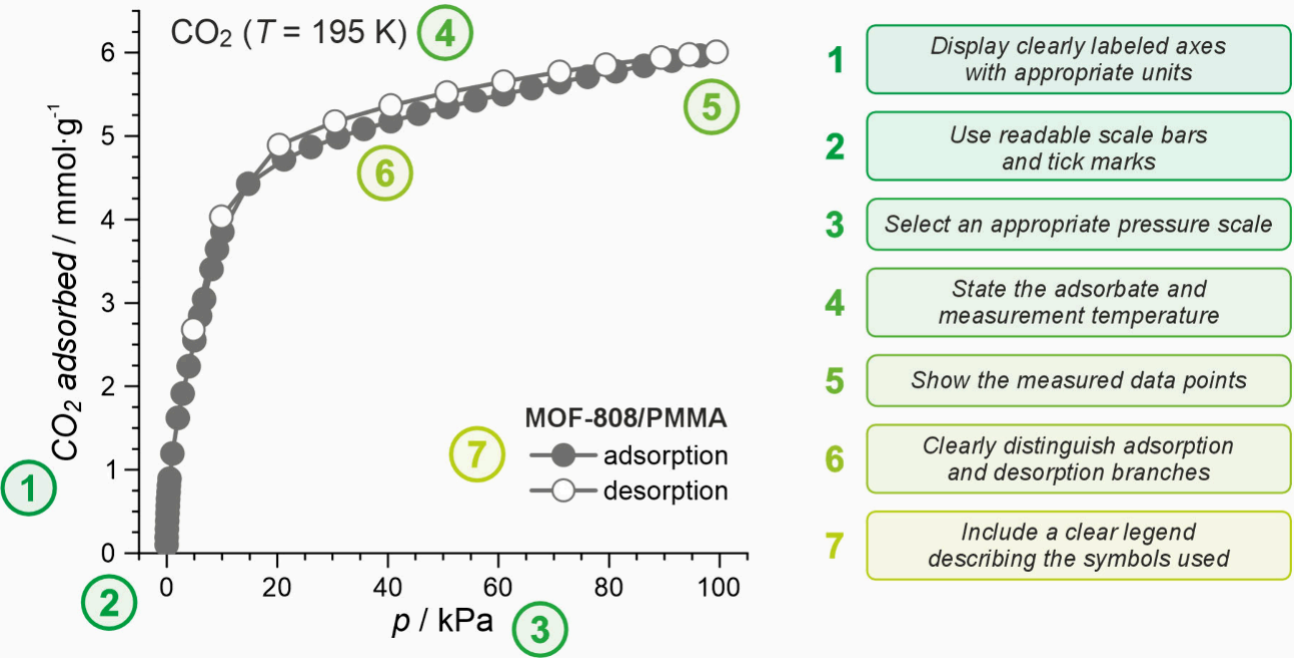
Graphical presentation of measured sorption isotherm of carbon dioxide (at 195 K) for a representative porous material (data reproduced from ref 53 for MOF/polymer hybrid, where PMMA is poly­(methyl methacrylate)). The annotations highlight recommended elements for clear presentation of adsorption isotherm data.

The adsorbed amount (*y*-axis) is commonly reported either as cubic centimeters of adsorptive gas at standard temperature and pressure (STP) per gram of outgassed (activated) sample (cm^3^(STP) g^–1^), or as millimoles of adsorbate per gram of outgassed adsorbent (mmol g^–1^). Although the values reported in cm^3^(STP) g^–1^ remain widespread in the literature, the use of this unit can introduce ambiguity because the definition STP is not universal. Different regional conventions and instrument software may adopt different default STP definitions, which can lead to inconsistencies if not explicitly stated. For this reason, it is advisible to use mmol g^–1^ as the primary unit, which is also in agreement with the IUPAC recommendations,^3^ and when cm^3^(STP) g^–1^ is reported the STP conditions should be explicitly defined. For clarity, the *y*-axis should generally start from 0 mmol g^–1^, and if detailed features at low loading require emphasis, enlarged or zoomed-in regions may be presented as separate panels rather than truncating the primary axis.

The pressure (*x*-axis) may be presented either as absolute pressure (*p*) or relative pressure (*p*/*p*_*0*_). When relative pressure is used, the value of *p*_*0*_ (the saturation vapor pressure of the pure adsorbate at the measurement temperature) should be clearly defined. This is particularly important for subcritical adsorbates such as N_2_ at 77 K or Ar at 87 K, where *p*_*0*_ corresponds to the saturation vapor pressure under experimental conditions. The usage of both linear and logarithmic scales can be appropriate depending on the studied material. For microporous MOFs, an additional representation of adsorption isotherm in a logarithmic pressure scale is strongly recommended to better visualize steep uptake at very low relative pressures (*p*/*p*_*0*_ < 10^–4^).

Reported isotherms should always be presented with the individual experimental points clearly visible in the plot. Displaying only a smoothed spline or interpolated curve is strongly discouraged as oversmoothing can obscure experimental artifacts such as insufficient equilibration, pressure instability, leaks, or stepwise uptake phenomena. Adsorption and desorption branches should be clearly distinguishable, which is typically afforded by using the filled and open symbols. When multiple materials or measurements are compared in a single figure, variation in symbol shape and color is recommended to ensure clarity also in grayscale reproduction.^54^ Finally, it is a good practice to also state directly in the figure the type of the adsorbate and temperature of the measurement (Figure 4).

### 4.2 Availability and Sharing of Raw Data and Metadata

Although adsorption isotherms are commonly presented in a graphical form, reporting data exclusively as figures limits reproducibility and quantitative reanalysis. At the same time, digitization of plotted data introduces uncertainty and may propagate errors in comparative studies or meta-analyses.^55^ Therefore, complete numerical isotherm data should be made available alongside the graphical representation, and it is strongly encouraged that adsorption studies include the complete numerical data sets either as part of the Supporting Information or deposited in an open data repository. Besides numerical values alone, the availability of metadata is equally important to ensure reproducibility. This may include information such as the mass of the outgassed sample, activation conditions, temperature control, equilibration criteria, instrument type, and related experimental details.

In recognition of these challenges, the adsorption community has undertaken efforts to standardize sorption data reporting.^56^ One outcome of these efforts is the development of the adsorption information file (AIF) format, which is designed to store not only numerical sorption data but also comprehensive metadata describing instrumentation, experimental conditions, and sample preparation.^55^ The AIF format supports the implementation of the FAIR data principles.^57^ Detailed recommendations on best practices for reporting experimental and simulated adsorption data, including guidance on the use of the AIF format, have been comprehensively described by Siderius et al.^44^ We strongly encourage readers to consult that work for practical instructions and examples of standardized reporting.

### 4.3 Data Analysis of Adsorption Isotherms

A measured adsorption isotherm provides quantitative information about the textural properties of the adsorbent. From a single isotherm, parameters such as the total pore volume, specific surface area, and pore size distribution can be derived. These quantities are commonly regarded as primary textural descriptors when characterizing porous materials. In practice, such analyses are most often performed by using nitrogen adsorption at 77 K or argon adsorption at 87 K. Beyond structural characterization, low-pressure isotherms measured at multiple temperatures can also be used to evaluate functional properties, most notably, the isosteric heat of adsorption of a given adsorbate. In the following sections, we briefly outline how these parameters can be determined from experimental isotherms and highlight common pitfalls, with particular attention to challenges frequently encountered in studies of MOFs. It should be emphasized that the calculated parameters are model dependent and should not be interpreted as intrinsic material constants.

#### 4.3.1 Pore Volume

The total pore volume (*V*_*p*_) of a mesoporous material is commonly derived from an adsorption isotherm at high relative pressure. This calculation assumes that the pores are filled with adsorbate in the bulk liquid state at the measurement temperature (by applying the Gurvich rule).^45^ In practice, *V*_*p*_ is calculated from the amount adsorbed at a relative pressure close to unity (commonly in the region *p*/*p*_*0*_ = 0.95–0.99 for N_2_ at 77 K) using the liquid molar volume of the adsorbate. For mesoporous materials showcasing a Type IV isotherm, a reliable estimate is typically obtained when the isotherm displays a well-defined plateau at high relative pressure (Figure 5b).

**Figure 5:**
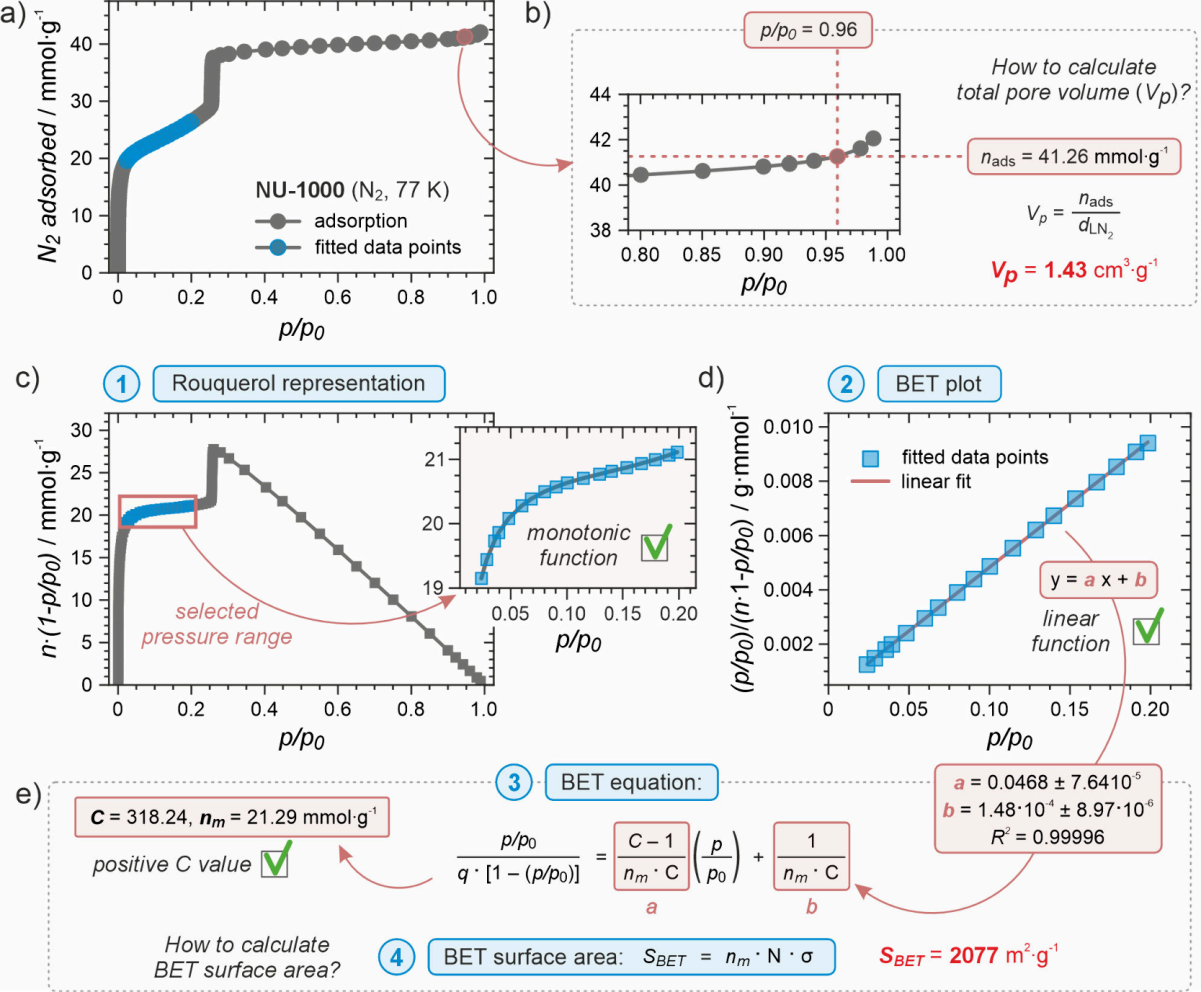
(a) Nitrogen adsorption isotherm measured at 77 K for a representative Zr-based MOF (data reproduced from ref 65 for NU-1000, raw data included in the Supporting Information). (b) Example of the selection of single data point for the total pore volume (*V*_*p*_) calculation; *n*_ads_ is specific amount of N_2_ adsorbed, *d*_LN2_ is density of liquid nitrogen (28.84 mmol cm^–3^). (c) Rouquerol representation of the measured isotherm with the zoomed region of fitted data points. (d) BET plot with linear fitting for selected pressure range used for analysis. (e) Example of BET surface area calculation from the fitted data, where *q* is the amount of N_2_ adsorbed at given pressure, *n*_*m*_ is monolayer capacity, *N* is Avogadro’s number (6.022 × 10^23^ mol^–1^) and σ is the cross-sectional area of the adsorbate molecule (0.162 nm^2^ for N_2_).

For practical considerations, the careful selection of the data point used for *V*_p_ calculation is essential. The chosen pressure should correspond to the plateau region of the adsorption isotherm rather than the steep uptake near *p/p*_0_ value close to 1. This sharp increase in uptake may result, for example, from the condensation of the adsorbate in the sample cell or from the presence of macropores or interparticle voids, which can lead to an overestimation of the pore volume (Figure 5c). Conversely, incomplete activation reduces the apparent pore volume. An example of total pore volume calculation from a data point selected near saturation pressure (*p*/*p*_0_ = 0.96), based on N_2_ adsorption measured at 77 K for Zr-based NU-1000, is shown in Figure 5b.

Experimental values of *V*_p_ can be compared with values computed based on the known crystal structure of analyzed material (e.g., using tools such as Zeo++^58^ or PoreBlazer^59^). In this case, proper selection of the probe molecule and the assessment of the *accessible* pore volume are important factors to consider.^60^ Because pore volume is typically derived from a single point or narrow pressure range, it is sensitive to data selection. The relative pressure used for the calculation, the adsorbate, and the temperature should therefore be clearly reported. When possible, the reproducibility across repeated measurements should be verified.

#### 4.3.2 BET Surface Area

The Brunauer–Emmett–Teller (BET) method remains the most widely used approach for estimating the specific surface area of porous materials from measured adsorption isotherms. The method is based on a multilayer adsorption model and yields the monolayer capacity, from which the surface area is calculated.^45^ In practice, the BET surface area is obtained by fitting the linearized BET equation within a selected relative pressure range (Figure 5d).

When applying the BET method, the most critical step is the selection of the fitting range, which should cover the initial linear region of the BET plot in the low relative pressure range and consist of at least 10 consecutive data points. The values of *n*_m_ and *C* can be obtained then from the linear regression (Figure 5d). Most commercial instruments provide automated BET calculations. However, the user should always verify the selected pressure ranges. Open-source alternatives such as pyGAPS^61^ and BETSI^62^ provide greater transparency, utilization of consistency criteria, and flexibility in the range selection and statistical evaluation.

After selecting a tentative fitting range, the result should be validated using the Rouquerol consistency criteria.^63,64^ These require a positive *C* constant, a monotonic increase of *n*(1 – *p*/*p*_0_), and agreement between the calculated monolayer capacity and the pressure range used for fitting (Figure 5c,d). Still, even when these criteria are fulfilled, multiple acceptable fitting ranges may exist. Thus, reporting a single surface area value without methodological details is insufficient. At a minimum, the selected relative pressure range used for fitting, the values of fitted parameters (the *C* constant and the monolayer capacity) should be provided. At the same time, excessive significant figures should be avoided. Because acceptable fitting ranges may produce noticeably different values, quoting surface areas with unrealistic precision can give a misleading impression of accuracy.

An example illustrating the practical application of BET analysis is shown in Figure 5, where the measured N_2_ adsorption isotherm (Figure 5a) is accompanied by the corresponding Rouquerol representation (Figure 5c) and the BET plot (Figure 5d). The raw adsorption data for this isotherm in AIF format are provided as Supporting Information to allow readers to reproduce the analysis and test different fitting ranges, e.g. by using available software tools such as pyGAPS.^61^ The presented example of BET calculation (Figure 5e) is based on selected data range of *p*/*p*_0_ = 0.02–0.20 (18 data points) which resulted in a BET surface area of 2077 m^2^ g^–1^. However, applying the automated BETSI^62^ procedure identifies a wider fitting region (27 data points in a range *p*/*p*_0_ = 0.01–0.25) that also satisfies all Rouquerol consistency criteria and yields a slightly higher BET surface area of *S*_*BET*_ = 2107 m^2^ g^–1^. This example illustrates that the BET surface area is a calculated parameter that depends on the selected fitting range and number of included data points. When many measured adsorption points satisfy the consistency criteria, multiple acceptable fitting regions may exist, leading to slightly different calculated surface areas.

Readers are encouraged to explore how selecting different fitting ranges influences the calculated BET surface area by monitoring changes in the C constant, monolayer capacity, and quality of the linear fit. Such hands-on analysis helps illustrate why reporting the selected *p*/*p*_0_ fitting range is essential for the proper interpretation and reproducibility of BET results. The strong dependence of the BET surface area on the fitting range has also been highlighted in a recent interlaboratory study, where different users analyzing the same isotherm reported significantly different BET surface areas.^62^

Nevertheless, simulation-based benchmarking studies showed that BET analysis can provide meaningful surface area estimations for microporous MOFs, provided that the fitting range is selected using consistency criteria.^66,67^ Additional caution is required when interpreting BET results for ultrahigh-porous MOFs, where overlap between monolayer formation and pore filling may lead to an overestimation of the monolayer capacity.^68–70^

#### 4.3.3 Pore Size Distribution

Pore size distribution (PSD) analysis provides information about the distribution of pore widths within a porous material. Unlike the pore volume or BET surface area, PSD is not obtained directly from experimental data but is derived through model-dependent analysis of the adsorption isotherm. Consequently, the PSD results are strongly influenced by the selected theoretical models.

In studies of MOFs, several models have been commonly employed. The Horvath–Kawazoe (HK)^71^ method is typically used for analysis of micropores, whereas the Barrett–Joyner–Halenda (BJH)^72^ model is applied for mesopore size distributions. More advanced approaches based on density functional theory (DFT)^73^ are also frequently used to describe more complex geometries, including NLDFT (nonlocal density functional theory),^74^ QSDFT (quenched solid density functional theory),^75^ and 2D-NLDFT models.^76^

It is worth mentioning that the DFT-based methods rely on precalculated theoretical adsorption models (so-called *kernels*) that describe adsorption in idealized pore geometries (e.g., slit, cylindrical, spherical pores). Many of these models were originally developed for carbon slit-pore systems and may not accurately describe the MOF environment, e.g., in regard to the presence of defects or framework flexibility. Moreover, PSD calculations are often difficult to reproduce from published data as the exact calculation procedure and model used are not always specified.

#### 4.3.4 Isosteric Heat of Adsorption

The isosteric enthalpy of adsorption (*Q*_st_) provides information on the strength of the interaction between an adsorbate and the surface of an adsorbent. This value is not a structural descriptor but reflects the energetic properties of the adsorption process. Importantly, the isosteric enthalpy is coverage-dependent and typically decreases with increasing loading as the highest-energy adsorption sites are occupied first. The indirect determination of *Q*_st_ requires preferably three isotherms measured at different temperatures where sufficient loadings can be achieved. For low-pressure adsorption studies in MOFs, isotherms are commonly measured at temperatures such as 273–293 K (e.g., for CO_2_) or 77 and 87 K (for H_2_ or D_2_). It is essential that all isotherms are measured on identically activated samples under comparable equilibration criteria.

Two approaches are commonly applied for calculating *Q*_*st*_, namely Clausius–Clapeyron (isosteric) method and virial analysis.^77^ In the isosteric method, the isotherms are first fitted with an appropriate adsorption model (e.g., Langmuir, dual-site Langmuir, or Freundlich-Langmuir). Pressures corresponding to identical loadings at different temperatures are then determined from the fitted curves, and *Q*_*st*_ is obtained from the slope of ln­(*p*) versus 1/*T* at constant loading. Alternatively, the virial method fits multiple isotherms simultaneously using a virial-type expression and derives *Q*_*st*_ directly from the temperature-dependent fitting coefficients.

A detailed practical guide to both methods, including implementation procedures, fitting strategies, and discussion of limitations, has been provided by Nuhnen and Janiak,^78^ and readers are encouraged to consult that work for computational details. When reporting *Q*_st_, the temperatures used, the calculation method, the fitting model (if applicable), and the loading range over which values were derived should be clearly specified. As with other derived parameters discussed in this Tutorial, the isosteric enthalpy of adsorption is model-dependent and should not be treated as an intrinsic material constant.

## 5 Theoretical Modeling of Sorption

In addition to the experimental sorption measurements discussed above, computational simulations can provide valuable complementary insight into adsorption processes in porous materials. In studies of MOFs, adsorption is commonly modeled using classical force fields, e.g., via grand canonical Monte Carlo (GCMC) simulations, which are less computationally expensive than *ab initio* methods. This approach is routinely used to interpret experimental isotherms,^47^ estimate adsorption enthalpies and site-specific interactions,^78^ and perform high-throughput screening of hypothetical or reported structures for targeted adsorptive properties.^79^ Several comprehensive overviews and perspectives of computational modeling in reticular materials have been reported recently.^80,81^ However, GCMC simulations should be interpreted with care, as the structural models are typically derived from idealized crystal structures and may not fully represent the material under experimental conditions, especially after guest removal, in the presence of framework flexibility, or when defects are present.

When reporting adsorption simulations, several key aspects must be clearly stated. The source of the structural model (experimental CIF, database entry, or hypothetical structure) must be specified, including any modifications, such as removal of guest molecules, treatment of disorder, or addition of missing hydrogen atoms. It is good practice to provide the modified structure used for simulations as a CIF or Cartesian coordinate file in the Supporting Information. Simulation parameters should include the pressure range, number of simulated points, equilibration, and production steps, and acceptance criteria.

## 6 Conclusions

Reliable sorption measurements are essential for evaluating the porosity and adsorptive properties of MOFs and for comparing the performance of the reported materials. This Tutorial provides a practical overview of the key steps involved in low-pressure volumetric gas adsorption experiments, including sample preparation, experimental setup, measurement procedures, and subsequent data analysis and reporting. Particular emphasis has been placed on aspects that are often insufficiently described in the literature but strongly influence the quality and interpretation of the adsorption data. To address this, we outlined recommended practices for activation protocols, measurement parameters, and data processing to facilitate more consistent and transparent reporting of sorption experiments. We hope that the practical guidance summarized herein will assist researchers in designing robust adsorption experiments and in reporting sorption data in a clear, consistent, and reproducible manner.

## Supplementary Material



## Notes

Two AIF files containing the sorption data corresponding to Figure 4 and Figure 5 are provided to allow readers to reproduce the analyses and explore the data-processing procedures discussed in this tutorial (ZIP)
